# Sleep Quality in Obesity: Does Adherence to the Mediterranean Diet Matter?

**DOI:** 10.3390/nu12051364

**Published:** 2020-05-10

**Authors:** Giovanna Muscogiuri, Luigi Barrea, Sara Aprano, Lydia Framondi, Rossana Di Matteo, Daniela Laudisio, Gabriella Pugliese, Silvia Savastano, Annamaria Colao

**Affiliations:** 1 Department of Clinical Medicine and Surgery, Endocrinology Unit, Federico II University “Federico II”, Via Sergio Pansini 5, 80131 Naples, Italy; luigi.barrea@unina.it (L.B.); saraaprano@hotmail.com (S.A.); lydiaframondi@gmail.com (L.F.); rossanadimatteo96@hotmail.com (R.D.M.); dani.laudisio@libero.it (D.L.); robiniapugliese@gmail.com (G.P.); sisavast@unina.it (S.S.); colao@unina.it (A.C.); 2 Centro Italiano per la cura e il Benessere del Paziente con Obesità (C.I.B.O), Federico II University, Via Sergio Pansini 5, 80131 Naples, Italy; 3 UNESCO Chair “Education for Health and Sustainable Development”, Federico II University, 80131 Naples, Italy

**Keywords:** Mediterranean diet, sleep quality, obesity, lifestyle, sleep disturbances, BMI

## Abstract

Obesity and unhealthy eating habits have been associated with sleep disturbances (SD). The Mediterranean diet (MD) is a healthy nutritional pattern that has been reported to be associated with better health and sleep quality. Thus, the aim of the study was to investigate whether adherence to the MD is associated with sleep quality in a population of middle-aged Italian adults. This cross-sectional study included 172 middle-aged adults (71.5% females; 51.8 ± 15.7 years) that were consecutively enrolled in a campaign to prevent obesity called the OPERA (Obesity, Programs of Nutrition, Education, Research and Assessment of the best treatment) prevention project that was held in Naples on 11–13 October 2019. Anthropometric parameters, adherence to the MD and sleep quality were studied. Overall, 50.6% of the subjects were good sleepers (the Pittsburgh Sleep Quality Index (PSQI) < 5) while 49.4% were poor sleepers (PSQI ≥ 5). Our results demonstrated that good sleepers, when compared to poor sleepers (*p* < 0.001) had significantly higher adherence to the MD as assessed by PREDIMED (Prevención con Dieta Mediterránea) score, lower BMI (body mass index) and waist circumference (WC). The higher PSQI, the higher the BMI (*p* < 0.001) and WC values (*p* < 0.001), thus suggesting that poor sleep was more common in subjects with obesity. In addition, a negative correlation between PSQI and the PREDIMED score (*p* < 0.001) was found. to the intake of the cluster of foods enclosed in the MD, rather than the intake of the single food, predicted PSQI. By performing a receiver operator characteristic (ROC) curve analysis, we determined a cut-off value at a PREDIMED score < 9 as the threshold for screening poor sleepers. In conclusion, good sleepers had lower BMI and WC and higher adherence to the MD than poor sleepers. PSQI was positively associated to BMI and WC while it was negatively associated to adherence to the MD. The consumption of the MD dietary pattern rather than the intake of a single nutrient has a beneficial effect on sleep quality. Hence, the assessment of sleep should be taken into account in the management of obesity and promoting adherence to the MD could be a tool to improve SD.

## 1. Introduction

Sleep disturbances (SD) are a common finding in obesity [1,2,3]. Obstructive sleep apnea (OSA) is the most prevalent type of obesity-related sleep disorder, which in turn, represents a risk factor for several health conditions. However, SD and OSA do not only affect subjects with severe obesity but also subjects with moderate obesity, thus suggesting that there could be further mechanisms that could contribute to disturbed sleep beyond the fat excess that causes recurrent narrowing and closure of the upper airway. Indeed, increased visceral adipose tissue has been reported to be responsible for the secretion of inflammatory cytokines that could disrupt the sleep-wake rhythm. Dietary intake has been reported to play a role in the regulation of sleep. In fact, high consumption of carbohydrate seems to negatively impact sleep quality [4]. A study performed in Japanese women reported an association between low intake of vegetables and fish and high intake of confectionary and noodles and poor sleep quality [5]. Some specific foods have been demonstrated to have sleep-promoting properties such as milk, fish, fruit and vegetables (i.e., kiwi fruit and tart cherry juice) [6]. A study investigating the association between macronutrient intake and SD reported that low protein intake (19% of energy from protein) was associated with difficulty maintaining sleep while low carbohydrate intake was only marginally associated, and high fiber intake positively influenced sleep [7]. It is already known that in many Western countries, cow’s milk has traditionally been considered a tranquilizing beverage with sleep-inducing properties and this is due to the presence of melatonin, which is a natural compound in cow’s milk, although its concentration increases significantly if cows are milked in darkness at night time. [8]. Similarly, food containing proteins rich in tryptophan are considered sleep-inducers since tryptophan is a precursor of the neurosecretory hormone melatonin [9]. Since the food components of the Mediterranean Diet (MD) could potentially have a beneficial effect on sleep quality, we hypothesized that subjects with high adherence to the MD had a better sleep quality than subjects with low adherence to the MD. Thus, we aimed to investigate whether adherence to the MD was associated with sleep quality in a middle-aged Italian population.

## 2. Materials and Methods

### 2.1. Study Design, Settings, Participants and Protocol

One-hundred seventy-two subjects aged 51.8 ± 15.6 years were recruited in a cross-sectional study during the OPERA (Obesity, Programs of Nutrition, Education, Research and Assessment of the best treatment) prevention project held in Naples on 11–13 October 2019 [10]. The OPERA prevention project is part of a larger program known as Campus 3S (Health, Sport and Solidarity), an “outdoor” health clinic that was established in Naples and has also moved to other locations in Italy (www.campussalute.it for details). Campus 3S aims to screen the health status of the general population by providing free consultations, visits, and diagnostics for and is held in different public squares of Italy. The OPERA prevention project is also a strategic project of the UNESCO Chair on “Health Education and Sustainable Development” (see https://www.unescochairnapoli.it/ for details). The OPERA prevention project was based on the organization of a medical, athletic, tasting and psychological path by which subjects with obesity were visited and given the right advices to start a weight loss program. All subjects gave their written informed consent to the study, which was carried out in agreement with the Helsinki declaration for human studies. Recruitment consisted of an informative talk in which the details of the research were explained to the subjects, and they were encouraged to take part to the study. Eligible participants for the study were adult subjects aged 18–75 years with normal liver, cardiopulmonary and kidney function as determined by interview. The exclusion criteria included type 1 diabetes, type 2 diabetes, cancer, alcohol or drug abuse, history of allergy or intolerance to food belonging to the MD, and the consumption of medications that could influence sleep quality or body weight. Subjects following a specific dietary regimen for any reason were also excluded from the study. No patient was suffering from anxiety or depression.

### 2.2. Sample Size Justification and Power

The sample size was determined using the software ClinCalc tool (www.clincalc.com) based on the results from Yu et al. [11]. A statistical power (1-β) of 95% and a level of significance (α) of 5% were considered, which resulted in a sample of 130 subjects being the necessary number for this study. We decided to round this up to 172 subjects in order to prevent any drop out.

### 2.3. Data Collection

Trained nutritionists assessed anthropometric parameters, asked standard questions including demographic informations and lifestyle habits. We defined “current smokers” when the subjects reported they smoked at least one cigarette per day and “non-current smokers” when the subjects reported they did not smoke.

#### 2.3.1. Anthropometric Parameters

Subjects dressed in light clothes and no shoes while the anthropometric parameters were assessed, as already reported [12,13]. The formula for body mass index (BMI) was the following: weight in kilograms divided by height in meters squared. A wall-mounted stadiometer was used to assess height. A calibrated scale was used to assess body weight. Waist circumference (WC) was measured to the closest 0.1 cm with a non-extensible tape. Grade I obesity was defined as a BMI ranging from 30 to 34.9 kg/m^2^, grade II obesity was defined as a BMI ranging from 35 to 39.9 kg/m^2^ and grade III obesity was defined as a BMI equal to or greater than 40.0 kg/m^2^ [14].

#### 2.3.2. Adherence to the Mediterranean Diet

As already reported [15,16], adherence to the MD was evaluated using the previously validated 14-item PREDIMED (Prevención con Dieta Mediterránea) questionnaire [16,17]. A qualified nutritionist administered the questionnaire during a face-to-face interview with all the enrolled subjects. Briefly, for each item, scores of 1 and 0 were assigned; PREDIMED score was calculated as the sum of all the scores attributed to the items. According to this score, the subjects were categorized as follows: 0–5, lowest adherence; score 6–9, average adherence; and score ≥ 10, highest adherence [16,17].

#### 2.3.3. Assessment of Sleep

Overall sleep quality was assessed with the Pittsburgh Sleep Quality Index (PSQI) [18]. Poor sleepeers were defined as subjects with PSQI score ≥ 5. Good sleepers were defined as subjects with PSQI score < 5.

### 2.4. Statistical Analysis

The data distribution was evaluated by the Kolmogorov–Smirnov test and data not normally distributed were normalized by logarithm. The chi square (χ^2^) test was used to determine the significance of differences in the frequency distribution of smoking habit, BMI categories, adherence to the MD and dietary components included in the PREDIMED questionnaire. Differences between poor and good sleepers were analyzed by Student’s paired t-test or ANOVA test followed by the Bonferroni post-hoc test. The correlations between study variables were performed using Pearson r correlation coefficients. In addition, a multiple linear regression analysis model (stepwise method), expressed as R^2^, Beta (β) and t, with PSQI score as the dependent variable, was used to estimate the predictive value of BMI, food items (use of extra virgin olive oil as main cooking lipid, extra virgin olive oil > 4 tablespoons, vegetables ≥ 2 servings/day, fruits ≥ 3 servings/day, red/processed meats < 1/day, butter, cream, margarine < 1/day, soda drinks < 1/day, glasses of wines ≥ 7/week, fish/seafood ≥ 3/week, commercial sweets and confectionery ≤ 2/week, tree nuts ≥ 3/week, poultry more than red meats) and PREDIMED score. Receiver operator characteristic (ROC) curve analysis was carried out in order to identify sensitivity and specificity, area under the curve (AUC), and IC, as well as cut-off values of the PREDIMED score in detecting poor sleepers. The test AUC for ROC analysis was also calculated and we entered 0.65 for AUC ROC and 0.5 for null hypothesis values. An Alfa α level of 0.05 (type 1 error) and a β level of 0.2 (type II error) were used as the cut-off values for statistical significance. Variables with a variance inflation factor (VIF) >10 were excluded in order to avoid multicollinearity. Values ≤ 5% were considered statistically significant. Data were collected and analyzed using the MedCalc^®^ package (Version 12.3.0 1993–2012 Mariakerke, Belgium).

## 3. Results

The study population consisted of 172 participants (71.5% females; 51.8 ± 15.7 years). In Table 1 the socio-demographic and anthropometric characteristics, adherence to the MD and PSQI score are reported. Obesity was present in most of the enrolled subjects. In particular, grade I obesity was detected in 58 subjects (33.7%), grade II obesity was found in 29 subjects (16.9%) while grade III obesity was detected in 20 individuals (11.6%). The mean PREDIMED score was 7.8 ± 2.2. Twenty-one (12.2%) subjects had low adherence, 110 (64%) had average adherence while 41 (23.8%) had high adherence to the MD. The mean score of PSQI was 6.49 ± 4.85. Eighty-seven (50.6%) subjects were good sleepers while 85 (49.4%) subjects were poor sleepers.

Good sleepers had significantly lower BMI and WC values compared to poor sleepers. The percentage of normal weight subjects was significantly higher in good sleepers while the percentage of subjects with obesity II and III was higher in poor sleepers. Poor sleepers had significantly lower PREDIMED scores and a higher percentage of subjects with average adherence to the MD. A significantly higher percentage of subjects with high adherence to the MD was found in the good sleeper category. No difference between the two groups were detected regarding age, gender and smoking habits (Table 2).

### Correlation Studies

**Figure 1:**
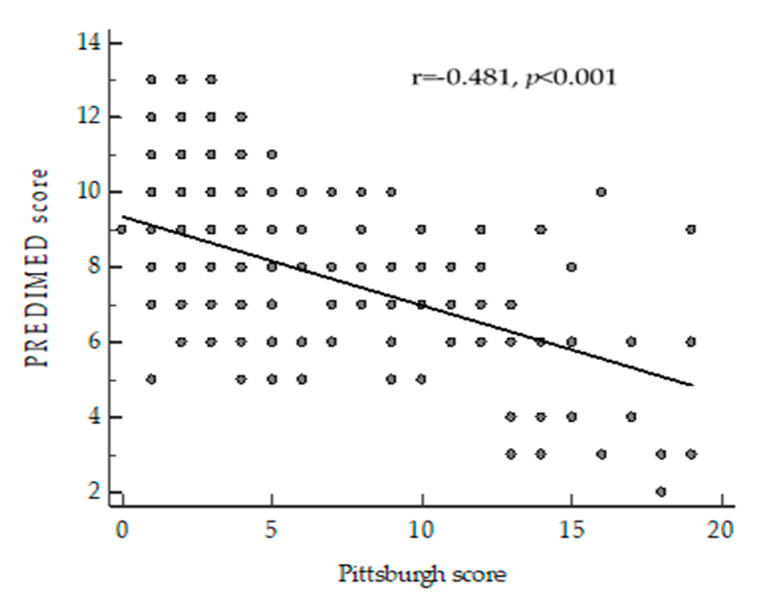
Correlation between PREDIMED score and PSQI adjusted for body mass index (BMI).

Correlation analyses were performed to assess the association of PSQI with age, anthropometric parameters and PREDIMED score (Table 3). PSQI was positively associated to BMI and WC while it was inversely correlated to PREDIMED score. The correlation between PSQI and PREDIMED score was kept (*p* < 0.001) after adjustment for BMI (Figure 1). No correlation was found between age and PSQI.

Table 4 reports the results of the bivariate proportional odds ratio model which was applied to assess the association between PSQI and food items in the PREDIMED questionnaire. Extra-virgin olive oil (EVOO), vegetable, fruit, fish, poultry, nuts and wine consumption were positively associated to PSQI while soda drinks, red meats, butter, cream, margarine commercial sweets and confectionery seemed to have a negative effect. To assess the most predictive factor of PSQI among the single PREDIMED items (use of extra virgin olive oil as the main cooking lipid, extra virgin olive oil > 4 tablespoons, vegetables ≥ 2 servings/day, fruits ≥ 3 servings/day, red/processed meats < 1/day, butter, cream, margarine < 1/day, soda drinks < 1/day, glasses of wine ≥ 7/week, fish/seafood ≥ 3/week, commercial sweets and confectionery ≤ 2/week, tree nuts ≥ 3/week, poultry more than red meats) and PREDIMED score, we performed a multiple linear regression analysis model that included these parameters. The PREDIMED score and BMI (*p* < 0.001) appeared to exert a powerful influence on PSQI (Table 5).

**Figure 2:**
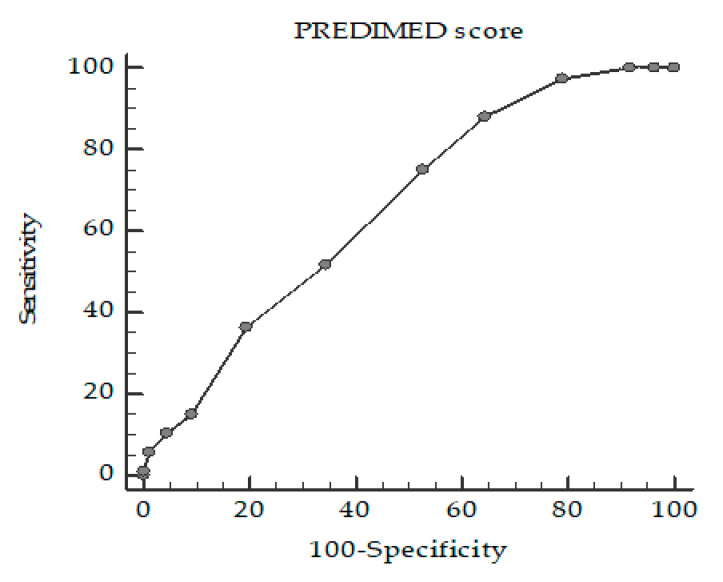
Receiver operator characteristic (ROC) for the cut-off value of PREDIMED score predictive of poor sleepers (PSQI ≥ 5).

A ROC curve analysis was then performed to determine the cut-off value of the PREDIMED score that is predictive of poor sleepers (PSQI ≥ 5). Specifically, PREDIMED scores < 9 (*p* < 0.001, AUC 0.65, standard error 0.04, 95% CI 0.58 to 0.72 (Figure 2) was identified as the thresholds for PSQI ≥ 5.

## 4. Discussion

In our study, 87 (50.6%) subjects were good sleepers while 85 (49.4%) subjects were poor sleepers. Good sleepers had a significantly higher adherence to the MD, lower BMI and waist circumference. PSQI positively correlated with BMI, WC and negatively correlated with PREDIMED score. The major determinant of PSQI was the cluster of food contained in the MD, rather than the single food. It is already well-known that obesity is associated with SD [1,2,3]. The main mechanism underlying this association is excessive adipose tissue that causes recurrent narrowing and closure of the upper airway [19]. However, this mostly concerns subjects with severe obesity although it has been reported that SD also affect subjects who are overweight or have grade I obesity, thus leading to the hypothesis that there could be some additional mechanism that relates fat excess and SD. Indeed, visceral adipose tissue could play an additional role in the pathogenesis of SD [20]. As is well-known, visceral adipose tissue plays an important role in the secretion of inflammatory cytokines, such as IL-1, IL-6 and TNF-α that are responsible for low-grade chronic inflammation [21]. It has been demonstrated that pro-inflammatory cytokines classified as “sleep-regulatory substances” could have a role in sleep regulation [22,23]. In particular, TNF-α and IL-1β have a circadian secretion, with the highest TNF-α and IL-6 secretion during the night (between 01:00 and 02:00), thus suggesting that they could be involved in the physiological regulation of sleep in both animals and humans [24] due to their effect on slow-wave sleep [25]. In subjects with obesity, TNF-α and IL-6 levels have been reported to be higher during the morning instead of the night and to be associated to BMI and SD [26]. In agreement with these findings, we found that WC, the most used indirect parameter of visceral adipose tissue, was higher in subjects with SD and was positively correlated with PSQI. All together these data suggest a hypothetical vicious circle involving obesity, pro-inflammatory cytokines levels and SD. Subjects with SD had a lower adherence to the MD in our study. The Seniors-ENRICA cohort study enrolled 1596 participants that were followed up for 2.8 years. In this population, high adherence to the MD at baseline was associated with a lower risk of reduced sleep duration and quality over the time of the study [27].

This previous study was performed in elderly subjects and we found similar findings in a middle-age population. In addition, Godos et al. [28] performed a cross-sectional study in 1936 individuals recruited in the urban area of Catania. They found that for each point increase in the MD score, individuals were 10% more likely to have adequate sleep quality [28].

However, the authors did not investigate if this association was mediated by a single food items included in the MD pattern or by the cluster of foods enclosed in the MD. We found that although subjects with SD were more prone to consume EVOO, vegetable, fruit, fish, poultry, nuts and wine and less prone to consume soda drinks, red meats, butter, cream, margarine commercial sweets and confectionery, the main determinant of sleep quality was the cluster of foods enclosed in the MD instead of individual components of the MD. The high content of polyunsaturated fatty acids (PUFA) and phytochemicals, such as polyphenols, in the MD have been reported to have a beneficial effect on inflammatory parameters that have been reported to have a negative impact on sleep duration and quality [29,30,31]. Most of the time, an increased adherence to a healthy nutritional pattern such as the MD is accompanied by an overall healthy lifestyle characterized by physical activity. Indeed, physically active subjects seem to sleep more and better than sedentary ones [32]. Although we did not assess physical activity, we could hypothesize that a sedentary lifestyle, which is often detected in subjects with obesity, could be an additional key player in SD Another interesting hypothesis concerns the effect of obesity-related hormonal disorders on sleep. Indeed, low growth hormone (GH) status is a common finding in obesity and it is considered to be an acquired functional defect; in fact, it has been demonstrated to be reversed after weight loss. A low GH status often seems to be associated with visceral obesity leading to the hypothesis that fat deposition at abdominal site could be one of the key players in GH secretion disorders in obesity [13]. Subjects with obesity that had high adherence to MD and in particular, proteins intake, showed a better GH status [13]. Furthermore, GH has also been reported to play an important role in regulating sleep [33]. Based on this, we hypothesize that the higher adherence to the MD that was detected in good sleepers could contribute, probably through protein intake, to stimulate the GH-IGF-1 axis, which is often diminished in obesity, thus contributing to normal sleep quality. Recently, the role of the quality of the nutritional pattern has been highlighted, as well as the role of energy intake and meal times in obesity-related SD [34,35]. In particular, there are epidemiological studies that have reported an association between obesity and chronobiological aspects [36,37,38]. One of the most interesting findings is that shift work represents an independent risk factor in the development of obesity [36]. Epidemiological studies have reported that shift work is associated with obesity, hypertriglyceridemia, low high-density lipoprotein, abdominal obesity, diabetes and cardiovascular disease [36,37]. All these issues seem to be due to the disrupted circadian rhythmicity of the melatonin profile detected in shift workers [38]. Therefore, the presence of proteins rich in tryptophan in the MD could be an additional mechanism that contributes to improve SD since tryptophan is a precursor of the neurosecretory hormone melatonin [9]. In addition, we identified the cut off of PREDIMED score < 9 to be associated with poor sleep. This suggests that sleep quality should be assessed when the PREDIMED score is < 9 because there is a high possibility of detecting SD. However, this study has certain limitations as well. The cross-sectional design can not reveal causality. Also, dietary intake and the assessment of SD were based only on self-reported data. The enrolled subjects did not complete a questionnaire on sleep disordered breathing and we did not have any information on whether subjects underwent previous sleep studies, therefore the degree to which the individuals had sleep apnea was not known. Thus, possible memory and reporting biases may affect the data to some extent. In addition, people with obesity often tend to misreport their food intake, and also, health-conscious people are more likely to participate in health surveys. Finally, no subjects self-reported anxiety and depression; in order to minimize this bias, we also asked for medications that they were taking to further ensure that they were not affected by these diseases and none of them reported taking any drugs.

## 5. Conclusions

In conclusion, we found that good sleepers had a higher adherence to the MD compared to poor sleepers. PSQI was positively correlated to BMI, waist circumference and negatively correlated to PREDIMED score. The main determinant of PSQI was the cluster of food contained in the MD, rather than single food items. Further research is needed to investigate the impact of nutritional patterns on sleep and to investigate the causality and mechanisms.

## Figures and Tables

**Table 1:** Lifestyle habits, anthropometric measurements, adherence to the Mediterranean diet (MD) and sleep assessment.

Parameters	Study Population n = 172
Age	51.8 ± 15.7 years
**Gender**	
Males	28.5%
Females	71.5%
**Smoking**	
Yes (n, %)	32, 55.0%
**Anthropometric measurements**	
Weight (kg)	84.6 ± 18.9
Height (m)	1.6 ± 0.09
BMI (kg/m^2^)	32.1 ± 6.3
Normal-weight (n, %)	18, 10.5%
Over-weight (n, %)	47, 27.3%
Obesity I (n, %)	58, 33.7%
Obesity II (n, %)	29, 16.9%
Obesity III (n, %)	20, 11.6%
WC (cm)	103.0 ± 16.0
**Adherence to the MD**	
PREDIMED score	7.8 ± 2.2
Low adherence to the MD (n, %)	21, 12.2%
Average adherence to the MD (n, %)	110, 64.0%
High adherence to the MD (n, %)	41, 23.8%
PSQI	6.49 ± 4.85
Good Sleepers (n, %)	87, 50.6%
Poor Sleepers (n, %)	85, 49.4%

Data are presented as mean ± standard deviation and/or percentages. A *p* value in bold type denotes a significant difference (*p* < 0.05).

**Table 2:** Age, gender, smoking, anthropometric characteristics and PREDIMED score of the study population according to PSQI score.

Parameters	Good Sleepers PSQI < 5) n = 87, 50.6%	Poor Sleepers (PSQI ≥ 5) n = 85, 49.4 %	*p* Value
Age (years) Gender	50.43 ± 15.7	53.28 ± 15.6	0.233
Males (n, %)	29, 33%	20, 24%	*p* = 0.21
Females (n, %)	58, 67%	65, 76%	
Smoking			
Yes (n)	11, 12.6%	21, 25%	*p* = 0.29
Anthropometric measurements			
Weight (kg)	77.79 ± 15.6	91.48 ± 19.3	**<0.001**
Height (m)	1.6 ± 0.09	1.6 ± 0.08	**0.034**
BMI (kg/m^2^)	28.9 ± 4.5	35.2 ± 6.1	**<0.001**
Normal-weight (n)	18	0	***p* < 0.001**
Over-weight (n)	28	19	*p* = 0.20
Obesity I (n)	34	24	*p* = 0.18
Obesity II (n)	7	22	***p* = 0.004**
Obesity III (n)	0	20	***p* < 0.001**
WC (cm)	97.01 ± 15.9	109.1 ± 13.7	**<0.001**
Adherence to the MD			
PREDIMED score	8.4 ± 2.2	7.1 ± 1.9	**<0.001**
Low adherence to the MD (n)	8	13	*p* = 0.32
Average adherence to the MD (n)	48	62	***p* = 0.02**
High adherence to the MD (n)	31	10	***p* < 0.001**

Data are presented as mean ± standard deviation and/or percentages. A *p* value in bold type denotes a significant difference (*p* < 0.05).

**Table 3:** Correlation of PSQI with age, anthropometric parameters and PREDIMED Score.

	PSQI Score	
Parameters	r	*p*-Value
Age (years)	0.092	0.228
BMI (kg/m^2^)	0.488	**<0.001**
WC (cm)	0.356	**<0.001**
PREDIMED Score	−0.522	**<0.001**

A *p* value in bold type denotes a significant difference (*p* < 0.05).

**Table 4:** Bivariate proportional odds ratio models performed to assess the association of PSQI with the dietary components included in the PREDIMED questionnaire.

Questions	OR	R^2^	95% IC	*p* Value
**Use of extra virgin olive oil as main culinary lipid**	0.84	0.06	0.75–0.93	**0.001**
**Extra virgin olive oil > 4 tablespoons**	0.89	0.05	0.83–0.96	**0.002**
**Vegetables ≥ 2 servings/day**	0.92	0.04	0.86–0.98	**0.007**
**Fruits ≥ 3 servings/day**	0.89	0.06	0.83–0.96	**0.002**
**Red/processed meats < 1/day**	0.88	0.08	0.82–0.94	**<0.001**
**Butter, cream, margarine < 1/day**	0.92	0.03	0.85–0.99	**0.024**
**Soda drinks < 1/day**	0.89	0.05	0.84–0.96	**0.002**
**Wine glasses ≥ 7/week**	0.88	0.04	0.79–0.97	**0.011**
**Legumes ≥ 3/week**	0.99	0.01	0.94–1.05	0.873
**Fish/seafood ≥ 3/week**	0.94	0.02	0.88–1.00	0.074
**Commercial sweets and confectionery ≤ 2/week**	0.92	0.03	0.87–0.99	**0.023**
**Tree nuts ≥ 3/week**	0.88	0.07	0.82–0.95	**0.001**
**Poultry more than red meats**	0.95	0.02	0.88–1.01	0.103
**Use of sofrito sauce ≥ 2/week**	0.99	0.01	0.94–1.07	0.983

A *p* value in bold type denotes a significant difference (*p* < 0.05).

**Table 5:** Multiple regression analysis models (stepwise method) with PSQI as the dependent variable was used to estimate the predictive value of the BMI, single PREDIMED items (use of extra virgin olive oil as main cooking lipid, extra virgin olive oil > 4 tablespoons, vegetables ≥ 2 servings/day, fruits ≥ 3 servings/day, red/processed meats < 1/day, butter, cream, margarine < 1/day, soda drinks < 1/day, glasses of wine ≥ 7/week, fish/seafood ≥ 3/week, commercial sweets and confectionery ≤ 2/week, tree nuts ≥ 3/week, poultry more than red meats) and PREDIMED score.

Parameters	Multiple Regression Analysis			
Model 1	R^2^	*β*	t	*p* Value
**PREDIMED score**	0.27	−0.52	−7.9	**<0.001**
**BMI (kg/m^2^)**	0.42	0.39	6.4	**<0.001**
Variable excluded: use of extra virgin olive oil as main cooking lipid, extra virgin olive oil > 4 tablespoons, vegetables ≥ 2 servings/day, fruits ≥ 3 servings/day, red/processed meats < 1/day, butter, cream, margarine < 1/day, soda drinks < 1/day, glasses of wine ≥ 7/week, commercial sweets and confectionery ≤ 2/week, tree nuts ≥ 3/week.				

A *p* value in bold type denotes a significant difference (*p* < 0.05).

## Abbreviations

SD	Sleep disturbance
MD	Mediterranean diet
BMI	Body mass index
PSQI	Pittsburg Sleep Quality Index
OSA	Obstructive sleep apnea
OPERA	Obesity, Programs of Nutrition, Education, Research and Assessment of the best treatment
WC	Waist circumference
PUFA	Polyunsaturated fatty acids
PREDIMED	Prevención con Dieta Mediterránea
EVOO	Extra-virgin olive oil
AUC	Area under the curve
VIF	Variance inflation factor
ROC	Receiver operator characteristic
GH	Growth hormone
